# Evaluating the relation between trait anxiety and stressful life events: implications for stress-induced depression vulnerability

**DOI:** 10.1192/j.eurpsy.2025.1320

**Published:** 2025-08-26

**Authors:** R. O. I. Font, M. C. Casanovas, A. D. A. Arnau, M. U. Sarachaga, V. S. Tomàs

**Affiliations:** 1 Psychiatry, Hospital de Bellvitge; 2 Psychiatry, Universitat de Barcelona, Barcelona; 3 Psychiatry, Hospital Parc Taulí, Sabadell, Spain

## Abstract

**Introduction:**

Major Depressive Disorder (MDD) significantly impacts global disability and quality of life. Some variables such as the trait anxiety and experiencing stressful life events (SLEs) are usually related to the MDD. However, the relationship between these variables in depression needs further investigation. Emerging research suggests the STAI-trait could be a nonspecific measure of negative effect and increase susceptibility to stress-induced depression.

**Objectives:**

This study assesses the State-Trait Anxiety Inventory (STAI) trait scores and SLEs, depressive symptoms, outcomed and functionality in MDD patients, with the hypothesis that STAI-trait may predispose individuals to stress-induced depression.

**Methods:**

A prospective observational study was conducted with 25 MDD patients recruited at Hospital Universitari de Bellvitge. The STAI-trait and SLE exposure were measured during the initial visit. Depression symptom and outcome variables were assessed in three sequential clinical evaluations.

**Results:**

Preliminary findings show a significant association between anxiety trait and SLEs, high STAI-trait scores correlated positively with increased SLEs. This, correlating with more severe MDD symptoms and a complex disease course.

**Conclusions:**

These findings support the notion of the STAI-trait as a possible mediator between life stressors and depression.

They highlight that increased STAI-trait anxiety might lead to greater vulnerability to stress and its potentially depressive effects, underscoring the need to consider this trait in clinical practice and the development of preventive strategies.

**Disclosure of Interest:**

None Declared

